# LC-MS and GC-MS Profiling of Different Fractions of *Ficus platyphylla* Stem Bark Ethanolic Extract

**DOI:** 10.1155/2022/6349332

**Published:** 2022-12-14

**Authors:** Madinat Hassan, Sunday Zeal Bala, Musa Bashir, Peter Maitalata Waziri, Ramlatu Musa Adam, Muhammad Abdullahi Umar, Priscilla Kini

**Affiliations:** ^1^Biology Department, Faculty of Science, Airforce Institute of Technology, Kaduna State, Nigeria; ^2^Biochemistry Department, Faculty of Basic Medical Science, Bayero University Kano, Kano State, Nigeria; ^3^Centre for Dryland Agriculture, Faculty of Agricultural Science, Bayero University Kano, Kano State, Nigeria; ^4^Biochemistry Department, Faculty of Science, Kaduna State University, Kaduna State, Nigeria; ^5^Biochemistry Department, Faculty of Science, Gombe State University, Gombe State, Nigeria; ^6^Department of Biochemistry and Biotechnology, Kwame Nkrumah University of Science and Technology, Kumasi, Ghana

## Abstract

The exploration of medicinal plants in traditional medicine for the treatment of diseases has been practiced for long, globally, because of its cultural acceptability, availability, and affordability. This study investigated the qualitative and quantitative estimation of phytochemicals present in *Ficus platyphylla* stem bark as well as determined the reducing power and antioxidant property of each fraction against DPPH and NO radicals. The study further elucidated the presence of possible compounds in different fractions (methanol, ethyl acetate, petroleum ether, and chloroform) of *Ficus platyphylla* stem bark (FPSB) extract using GC-MS, LC-MS, and FTIR techniques. Qualitative phytochemical analysis reveals the presence of phytochemicals: saponin, flavonoids, tannins, phenols, steroids, alkaloids, and glycoside in the ethanolic extract. The LC-MS study of methanol and ethyl acetate fractions reveals the presence of thirteen and three compounds, respectively. GC-MS analysis shows the presence of trans-13-octadecenoic acid as the main compound 38.07% and cis-vaccenic acid as the least compound (0.10%) in the petroleum ether fraction. The main compound in the chloroform fraction is 12-oleanen-3-yl acetate, (3. alpha.) with a peak area percentage of 49.25% and oleic acid been the least compound with 0.07% peak area. The FTIR analysis reveals that the fractions contain compounds with hydroxyl, aromatic, methyl, methylene, methyne, long aliphatic chain, ethers, ether-oxy, peroxides, etc. The analyzed fractions reveal compounds with potential pharmacological activity in the management of pathological conditions.

## 1. Introduction

Phytomedicine is a prominent form of traditional medicine, which has been in practice since time immemorial across the globe as a therapeutic approach to manage specific pathological conditions [1]. Phytomedicine has been a culturally acceptable procedure to ameliorate many ailments because of its affordability and availability. This involves the use of herbal materials that may contain whole plant or its parts, which is known to enclose certain components that may affect the changes in the human body. Thus, various plants have been exploited for numerous purposes in the life of mankind and animals, explicitly, as food for nutritional benefits and medicines for the treatment of diseases [2]. Plants synthesize numerous chemical components called phytochemicals. Phytochemicals are non-nutritive bioactive components that have disease-preventive properties [3]. They are basically classified into six major categories on the basis of their chemical structures and characteristics, namely carbohydrates, lipids, phenolics, terpenoids, alkaloids, and other nitrogen-containing compounds [4]. These categories are further divided via biogenesis to obtain alkaloids, saponins, glycosides, lignans, flavonoids, tannins, triterpenes, coumarins, carotenoids, etc., which acts individually or synergistically to exhibit a useful or harmful effect to the body [4]. These phytochemicals are known to perform various functions and have been established to possess myriad of biological activities ranging from antibacterial, antifungal, antiviral, antioxidant, anticancer, hepatoprotective, antitumor benefits, and so much more. Modern drug research utilizes ethnobotany to search for pharmacologically active components (phytochemical) in plants, in order to alleviate serious side effects associated with synthetic drugs.

*Ficus platyphylla* is described as a deciduous plant belonging to the *Moraceae* family and usually grows in the tropical regions of world, basically in West Africa. In Nigeria, it is widely grown in the Northern part of the country. The tree grows to a height of about 60 fts. In Hausa language, the tree is locally referred to as “Gamji” and belongs to the family of fig trees [5]. Traditionally, different parts of the plant such as the leaves, stem, bark, and root are believed by the Hausa communities in Northern Nigeria to be efficacious in the treatment of different ailments such as psychosis, inflammation, epilepsy, and depression [5]. However, the stem bark of *Ficus platyphylla* has been predominantly used in Hausa traditional folk medicine to manage convulsive disorders. Phytochemical study from previous research has confirmed the presence of flavonoids, tannins, and saponins in *Ficus platyphylla* stem bark [6]. *Ficus platyphylla* was reported to possess antioxidant, anti-inflammatory, anti-insomnia, hepatoprotective, wound healing, and analgesic activities [7, 8].

To ascertain the efficacy of a specific medicinal plant to manage specific ailments, one of the fundamental processes to undertake is extraction of phytochemicals contained in the plant. Plant extract usually contains several chemical constituents working synergistically to act as medicines (phytopharmaceuticals), toxins (phytotoxins), or both (phytopharmatoxins) [9]. Plant extracts can be obtained using selective solvent (polar, intermediate polar, and nonpolar) through various standard extraction procedures ranging from decoction, infusion, maceration, percolation, hot continuous extraction (Soxhlet), sonication, microwave-assisted extraction (MAE), supercritical fluid extraction, etc [10]. High content of polyphenolic components in medicinal plant has been related to their antioxidant effect that are vital in prevention of the development of age-related diseases, specifically those associated with oxidative stress [7]. The determination for a good procedure during extraction highly depends on the type of plant material, chemical properties of the solvent used, physical parameters of the experiment, and the intended use of final products [8]. The investigation of the therapeutic effect of plants is vital for the exploration of medicinal plants; however, the determination of the essential active ingredients in a plant material can be overwhelming. Therefore, the study qualitatively and quantitatively assessed the presence of phytochemicals, determined the antioxidant property of each fraction against DPPH and NO radicals as well as reducing power activity, and identified bioactive compounds present in four different fractions of *Ficus platyphylla* stem bark.

## 2. Materials and Methods

### 2.1. Collection of Plant Material

*Ficus platyphylla* (Moracea) stem bark was obtained from a village called Karau-Karau, located in Zaria, Kaduna state, Nigeria with GPS co-ordinates; 11.061516, 7.716590, on 24th November, 2019.

### 2.2. Identification of Plant Material

Identification and authentication of the plant material was carried out at the Biology Department, Ahmadu Bello University, Zaria, Kaduna State by the botanist Mr. U.S. Gallah. A sample with voucher number 7719 was deposited in the Herbarium.

### 2.3. Preparation of Plant Material

The stem bark of the plant was dried under shade for fourteen days and pulverized using a pestle in a mortar to fine powder. This was subsequently stored in a labelled airtight container.

### 2.4. Preparations of Crude Extract of Ficus platyphylla Stem Bark

Extraction was achieved using microwave-assisted extraction (MAE) as reported by Hossain et al. [11]. Briefly, 1000 g of pulverized *Ficus platyphylla* stem bark was dissolved in 3500 mL of 70% ethanol. The solution was microwaved for two minutes at 80°C. After cooling, the extract obtained was filtered using a Whatman filter paper (No. 41). The obtained filtrate was concentrated using a rotary evaporator.

### 2.5. Qualitative Phytochemical Screening

The phytochemical constituents of *Ficus platyphylla* stem bark (FPSB) was screened qualitatively for presence or absence of flavonoids (lead acetate test), phenols (ferric chloride test), saponin (foam test), tannin (gelatin test), alkaloids (Dragendorff's reagent), cardiac glycosides, anthraquinones, and terpenoids using methods described by Saeed et al. [12]. Steroid was screened for using the method described by Ezeonu and Ejikeme [13].

### 2.6. Test for Alkaloids

Ethanolic extract of *Ficus platyphylla* stem bark (0.4 g) was stirred with 8 mL of 1% HCl, and the mixture was warmed and filtered. The filtrate (2 mL) was treated separately with potassium bismuth (Dragendroff's reagent). Turbidity or precipitation with either of this reagent was taken as evidence for existence of alkaloids.

### 2.7. Test for Saponins

Ethanolic extract of *Ficus platyphylla* stem bark (20 mg) was boiled in 20 ml of distilled water for 5 min and filtered. The filtrate (10 mL) was mixed with 5 mL of distilled water. The obtained mixture was vigorously shaken for froth formation. Three drops of olive oil was mixed with froth, shaken vigorously, and observed for emulsion development.

### 2.8. Test for Terpenoids

Presence of terpenoids was screened for using 5 mL (1 mg/mL) of ethanolic extract of *Ficus platyphylla* stem bark and mixed with 2 mL of chloroform, followed by 3 mL of concentrated H_2_SO_4_. A reddish-brown coloration of the interface confirmed the presence of terpenoids.

### 2.9. Test for Anthraquinones

Ethanolic extract of *Ficus platyphylla* stem bark (200 mg) was boiled with 6 mL of 1% HCl and filtered. The filtrate was shaken with 5 mL of benzene, filtered, and 2 mL of 10% ammonia solution was added to the filtrate. The mixture was shaken and the presence of a pink, violet, or red colour in the ammoniacal phase indicated the presence of free hydroxyl anthraquinones.

### 2.10. Cardiac Glycosides

Presence of cardiac glycoside was screened for using 5 mL (10 mg/mL in methanol) of ethanolic extract of *Ficus platyphylla* stem bark mixed with 2 mL of glacial acetic acid (having a drop of FeCl_3_ solution). To the mixture obtained, 1 mL of concentrated H_2_SO_4_ was added to form a layer. The presence of brown ring of the interface indicated deoxy sugar characteristic of cardiac glycosides.

### 2.11. Test for Flavonoids

Ethanolic extract of *Ficus platyphylla* stem bark (50 mg) was suspended in 100 mL of distilled water to get the filtrate. A diluted ammonia solution (5 mL) was added to 10 ml of obtained filtrate, followed by few drops of concentrated H_2_SO_4_. Presence of flavonoids was confirmed by yellow coloration.

### 2.12. Test for Tannins

Ethanolic extract of *Ficus platyphylla* stem bark (50 mg) was boiled in 20 mL of distilled water and filtered. A few drops of 0.1% FeCl_3_ was added to the filtrate and observed for colour change. A brownish green or a blue-black coloration was taken as evidence for the presence of tannins.

### 2.13. Test for Steroids

Ethanolic extract of *Ficus platyphylla* stem bark (0.30 g) was reconstituted in 20 mL of ethanol, the mixture was extracted for 2 hours. 5 mL of the extract was added to 2 mL acetic anhydride followed with 2 mL of concentrated H_2_SO_4_. A violet to blue or green colour change in sample(s) indicates the presence of steroids.

### 2.14. Test for Phenols

Ethanolic extract of *Ficus platyphylla* stem bark was treated with 3-4 drops of ferric chloride solution. Formation of bluish-black colour indicated the presence of phenols.

### 2.15. Preparations of Fractions from Ethanolic Extract of *Ficus platyphylla* Stem Bark

The crude extract was partially purified by sequential extraction using different solvents with different (increasing) polarity starting from petroleum ether, to chloroform, ethyl acetate, and finally methanol. The fractionation was done using Soxhlet apparatus (temperature = 40°C, time = 2-3 hours) as described by Hossain et al. [11]. This was done by tying the dried ethanolic extract with a muslin cloth and then inserted into the thimble of the Soxhlet apparatus. The petroleum ether was poured in the distillation flask below and allowed to heat at 40°C. The vapour resulting from the distillation flask condenses in the thimble-holder and dissolves the dried crude extract tied inside the thimble. As soon as the condensed petroleum ether in the extraction chamber reaches the overflow level, the solution in the thimble-holder was aspirated by a siphon and returns in the distillation flask. This was continuously done, until all compounds soluble in that solvent was extracted out. The sample (dried crude extract) was then removed, untied, and dried under air. The aforementioned procedure was done for chloroform, ethyl acetate, and methanol. The obtained fractions of petroleum ether, chloroform, ethyl acetate, and methanol were concentrated via rotary evaporator, and the dried residues of each fraction were subjected to quantitative phytochemical screening, antioxidant assays, and phytochemical identification techniques using standard procedures. The ethyl acetate and methanol fraction were subjected to LC-MS analysis while the petroleum ether and chloroform were subjected to GC-MS analysis on the basis of the polarity of solvent and target compounds.

### 2.16. Percentage Yield of Extract

The percentage yield of ethanolic extract and other fractions of *Ficus platyphylla* stem bark was obtained after drying in the water bath for 48 h at 40°C and was calculated as

(1) $$\begin{array}{c} Yield\;of\;extract\left(\%\right)=\frac{\left(Weight\;of\;extract\right)}{\left(Weight\;of\;dry\;plant\;before\;extraction\right)}\times 100\text{.} \end{array}$$

## 3. Quantitative Phytochemical Screening of Petroleum Ether, Chloroform, Ethyl Acetate and Methanol Fraction of *Ficus platyphylla* Stem Bark

### 3.1. Total Phenol

The total phenolic content of the obtained fractions was spectrometrically analyzed using the Folin−Ciocalteu method [14]. Briefly, 100 *μ*L of each fraction dissolved in methanol (0.2, 0.4, 0.6, 0.8 and 1.0 mg/mL) and standard gallic acid was mixed with 2 mL of 2% (w/v) sodium carbonate solution. The mixture was incubated for 5 min, and afterwards, 100 *μ*L of Folin−Ciocalteu reagent was added. The mixture was kept for 30 min at 25°C for colour development. Absorbance was subsequently measured at 750 nm using a spectrophotometer. Results were expressed as mg/g of gallic acid equivalents (GAE) of dried *Ficus platyphylla* stem bark fraction.

### 3.2. Total Flavonoid

The total flavonoid content of each fraction was determined using the aluminium chloride colorimetric assay with slight modifications reported by Abdulqayoom et al. [15], and quercetin was used as a standard to construct the calibration curve. Quercetin (25 mg) was dissolved in 25 mL of aqueous ethanol (1 mg/mL stock solution) and then diluted to 0.2, 0.4, 0.6, 0.8 and 1.0 mg/mL with ethanol. About 20 *μ*L each of the different fractions (0.1 g in 10 mL aqueous ethanol) and standard solution (0.2 to 1.0 mg/mL) were mixed with 15 *μ*L of sodium nitrite (0.5% NaNO_2_, w/v) solution separately in a 96 well plate and incubated for 6 min at room temperature (25°C). Thereafter, 15 *μ*L of (1% AlCl_3_, w/v) solution was added to each reaction well and allowed to stand for further 6 min before the addition of 80 *μ*L of sodium hydroxide (0.4% NaOH, w/v) to each well. The mixtures were incubated for another 15 min at room temperature (25°C), and absorbance was taken at 510 nm. The amount of flavonoid was calculated from linear regression equation obtained from the quercetin standard calibration curve. The flavonoid content was calculated as mean ± SD (*n* = 3) and expressed as mg/g of quercetin equivalent of dried *Ficus platyphylla* stem bark fraction.

## 4. Antioxidant Assays of Petroleum Ether, Chloroform, Ethyl Acetate and Methanol Fraction of *Ficus platyphylla* Stem Bark

### 4.1. 2,2-Diphenyl-1-picrylhydrazyl (DPPH) Radical Scavenging Activity Assay

The DPPH radical scavenging assay was conducted in accordance to the method reported by Zhu et al. [16]. Briefly, 2 mL of DPPH solution (0.1 mM, in methanol) was mixed with 2 mL of the four different extracts at varying concentrations of 20, 40, 60, 80, 100, 120, and 140 *μ*g/mL. The reaction mixture was shaken and incubated in the dark at 25°C for 30 min. The absorbance was read at 517 nm against the blank. Ascorbic acid was used as positive controls and prepared in a similar manner, as for the test samples. The inhibition of the DPPH radical by the sample was calculated based on the following formula:

(2) $$\begin{array}{c} \%\;Inhibition=\frac{\left(Absorbance\;of\;control\right)-\left(Absorbance\;of\;sample\right)}{\left(Absorbance\;of\;control\right)}\times 100\text{.} \end{array}$$

The half-maximal inhibitory concentration (IC_50_) was measured to indicate the concentration required to inhibit DPPH radicals by half, and it was derived from the graph equation obtained from plot of respective concentration of standard ascorbic acid and each fraction (petroleum ether, chloroform, ethyl acetate, and methanol) against their obtained percentage inhibition.

The IC_50_ value is calculated from the graph equation *Y* = *MX* − *C*, Where *Y* = 50, *X* = IC_50_ value, *M* = coefficient of *X* and *C* = constant.

### 4.2. Nitric Oxide Inhibition Assay

The assay was conducted as reported by Fadzai et al. [17]. The stock of each fraction and ascorbic acid was prepared (100 mg/mL) in methanol. These were then serially diluted to make concentrations of 20, 40, 60, 80, 100, 200, and 400 *μ*g/mL. Griess reagent was prepared by mixing equal amounts of 2% sulphanilamide in 5% phosphoric acid and 0.1% naphthyl ethylenediamine dihydrochloride immediately before use. A volume of 50 *μ*L of 10 mM sodium nitroprusside (0.29 g/100 mL) in 0.1 M phosphate buffered saline was mixed with 50 *μ*L of the different concentrations prepared in 96 well plate and then incubated at 25°C for 180 min. 100 *μ*L of Griess reagent was added to the solution mentioned above. A control sample without the extracts but with an equal volume of methanol was prepared in a similar manner as was done for the test samples. The absorbance was measured at 542 nm. The percentage nitrite radical scavenging activity of the respective fractions and ascorbic acid was calculated using the following formula:

(3) $$\begin{array}{c} \%\;Inhibition=\frac{Absorbance\;of\;Control-Absorbance\;of\;Sample}{Absorbance\;of\;Control}\times 100\text{.} \end{array}$$

### 4.3. Reducing Power Assay

According to the method reported by Nayan et al. [18], the aliquots of various concentrations of ascorbic acid (standard) and test samples (20 to 400 *μ*g/mL) were dissolved in 1 mL of deionized water, which was then mixed with 2.5 mL of (pH 6.6) phosphate buffer and 2.5 mL of (1%) potassium ferricyanide. The mixture was incubated at 50°C in water bath for 20 min after cooling. Aliquots of 2.5 mL of (10%) trichloroacetic acid were added to each of the mixture, which was then centrifuged at 3000 rpm for 10 min. The upper layer of 2.5 mL of solution was mixed with 2.5 mL of distilled water and a freshly prepared 0.5 mL of (0.1%) ferric chloride solution. The absorbance was measured at 700 nm. A blank was prepared without adding extract.

### 4.4. Identification of Ethyl Acetate and Methanol Fraction of FPSB Using Liquid Chromatography Mass Spectroscopy (LC-MS)

The LC Waters e2695 separation module with W2998 PDA coupled to ACQ-QDA MS was used for this study. The ethyl acetate and methanol fraction of FPSB was analyzed using an LC tandem MS as described by Piovesana et al. with slight modification [19]. Reconstitution of the fractions was done using methanol. The filtration was carried out using polytetrafluoroethylene (PTFE) membrane filter (0.45 *μ*m size). Ten microliters (10 *μ*L) of the filtrate were introduced (injected) into the liquid chromatographical system. This was separated on Sunfire C_18_ 5.0 *μ*m 4.6 mm × 150 mm column. The run was performed at a flow rate of 1.0 mL/min. The temperature of the column was fixed at 25°C with a 0.1% formic acid mixed in water serving as solvent A in the mobile phase. Also, formic acid (0.1%) was mixed with acetonitrile serving as solvent B within the gradient. A ration of 95 : 5 (A : B) was maintained for another 1 minute. 5 : 95(A : B) for 13–15 minutes, 95 : 5 (A : B) to 17, 19 and 20 minutes. Setting the PDA detector at 210–400 nm, 1.2 nm resolution and10 points/sec sampling rate. Scan range from *m*/*z* 100–1250 was used to acquire mass spectra maintaining the following settings: ESI source in both positive and negative ion modes; 600°C probe temp, 10 mL/min flow rate, and 45 psi nebulizer gas. The MS was set with a fragmentation voltage of 125 V in an automatic mode. Processing of data generated was done using Empower 3. The following information was used in the identification of the various compounds present in the plant material, retention time (tR), elution order, fragmentation pattern, and Base *m*/*z*.

### 4.5. Gas Chromatography-Mass Spectroscopy Analysis of Petroleum Ether and Chloroform Fractions

The petroleum ether and chloroform fractions were analyzed using GS-MS as described by Konappa et al. [20]. The GC system (PerkinElmer Clarus 600) was close-fitting a Rtx-5MS capillary column. A constant flow rate of 1.0 mL/min was maintained with helium (99.99%) as the carrier gas. The method used for the detection of the GC-MS spectral lines was the ionization energy method, with ionization energy of 70 eV (electron volt) and a 0.2 seconds scan time with a ranging fragment from 40 to 600 m/*z*, with one microliter (1 *μ*L) injection quantity (split ratio 10 : 1) and a temperature maintained at 250°C. However, the column oven temperature was at 50°C running for 3 minutes and 10°C temperature increase per minutes up to 280°C, with a final temperature of about 300°C for 10 minutes. Retention time per minute, peak area, peak height, and spectral lines patterns were used to identify the components present in the sample plant materials when compared with spectral lines from the database of authenticated compounds stored in the National Institute of Standards and Technology (NIST) library [21].

### 4.6. Fourier-Transform Infrared Spectroscopy (FT-IR Agilent Carry 630)

Protocol for FTIR spectroscopy was done as reported by Satapute et al. [22] which involves the encapsulation of 10 mg of the dried petroleum ether, chloroform, ethyl acetate, and methanol fractions in hundred milligrams (100 mg) of potassium bromide (KBr) pellet. This was carried out to prepare the translucent sample discs. The samples (400 to 4000 scan range) were loaded into the FTIR spectroscope, with 4 cm^−1^ resolution for each fraction.

### 4.7. Statistical Analysis

Analyses were carried out in triplicates using SPSS version 16 (IBM Inc. USA) and values were expressed as mean ± standard deviation. One-way analysis of variance (ANOVA) was used to determine the level of significance at 95% confidence interval followed by Tukey's multiple comparison test. The resolution quality of figures [2–5] was harnessed using an Anguage digitizer to obtain the data points of both axis into an excel table and R-analytic software was used in plotting the figures.

## 5. Results

The result in Table 1 reveals the phytochemical constituent of *Ficus platyphylla* stem bark ethanolic extract (70% aq.).

### 5.1. Total Phenol and Total Flavonoid

The methanol fraction of *Ficus platyphylla* stem bark contained the highest flavonoid content at concentration of 527.38 mg compared to ethyl acetate (512.74 mg), chloroform (274.89 mg), and petroleum ether fractions (126.49 mg). The highest total phenol content was observed in methanol fraction with a concentration of 92.46 mg compared to ethyl acetate (86.63 mg), chloroform (59.31 mg), and petroleum ether (7.02 mg) fractions, respectively. The abovementioned details can be found in Table 2.

### 5.2. Antioxidant Activity

**Figure 1:**
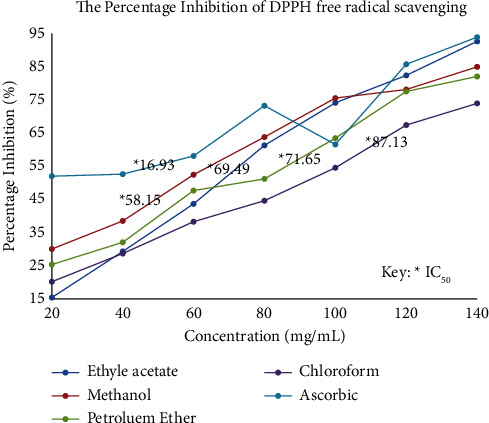
The percentage DPPH-free radical scavenging activity obtained from different fractions of ethanolic extract of *Ficus platyphylla* stem bark.

Methanol fraction reveals the highest scavenging activity against DPPH radicals (84.90 ± 0.05) at concentration of 140 *μ*g/mL with IC_50_ of 58.15 *μ*g/mL, although less than the standard-ascorbic acid (93.85 ± 0.05) at concentration of 140 *μ*g/mL with IC_50_ of 16.93 *μ*g/mL (Figure 1). Methanol fraction also showed a strong scavenging activity against NO (92.42 ± 0.08) at 20 *μ*g/mL compared to other fractions as shown in the table given below, although being lower than ascorbic acid (96.48 ± 0.05) at 20 *μ*g/mL (Table 3). The reducing power assay reveals methanol fraction activity to exhibit high reduction of Fe^3+^ to Fe^2+^ (1.33 ± 0.03) compared to other fractions as shown in Table 4.

### 5.3. LCMS Analysis of Methanol and Ethyl Acetate Fractions of *Ficus platyphylla* Stem Bark (FPSB)

**Figure 2:**
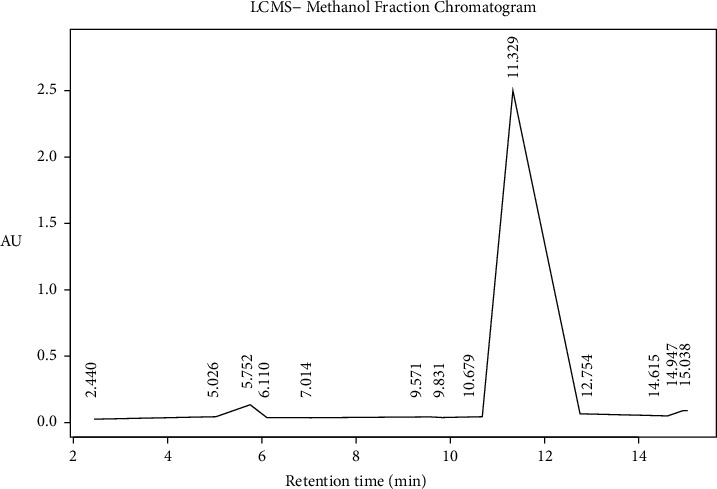
LC-MS chromatogram of methanol fraction of FPSB ethanolic extract.

**Figure 3:**
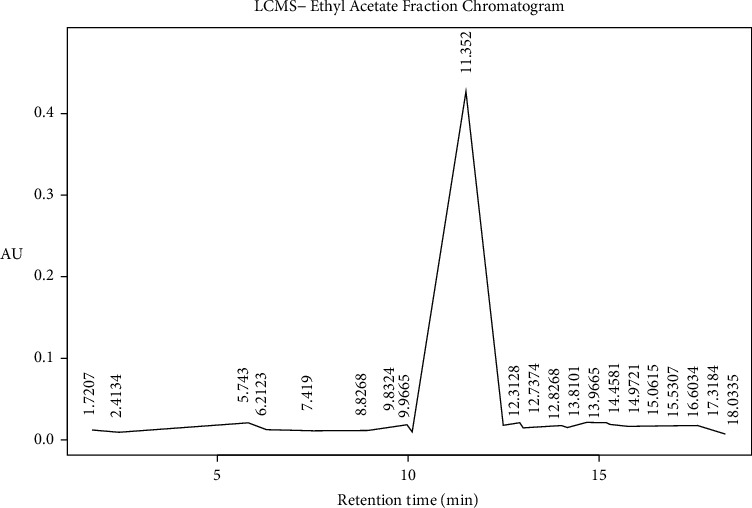
LC-MS chromatogram of ethyl acetate fraction of FPSB ethanolic extract.

**Figure 4:**
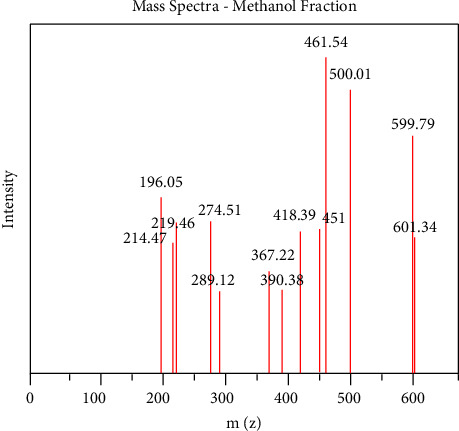
Mass spectra of methanol fraction of FPSB ethanolic extract.

**Figure 5:**
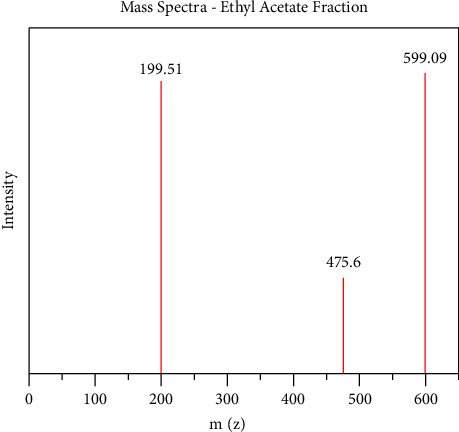
Mass spectra of ethyl acetate fraction of FPSB ethanolic extract.

LC-MS analysis was conducted for methanol and ethyl acetate fraction of FPSB on the basis of polarity of the solvent used. The LC-MS analysis reveals the retention time of the possible compounds as shown in Figures 2 and 3 and the fragmentation pattern reveal the relative abundance of the identified compounds as shown in Figures 4 and 5. Tables 5 and 6 show the identified compounds in methanol and ethyl acetate fraction, respectively.

### 5.4. GC-MS Analysis of Petroleum Ether and Chloroform Fraction

The petroleum ether fraction reveals thirty-four compounds and eighteen compounds in the chloroform fraction as shown in Tables 7 and 8, respectively.

### 5.5. FTIR Analysis of Ethyl Acetate Fraction of Ethanolic Extract of *Ficus platyphylla* Stem Bark

**Figure 6:**
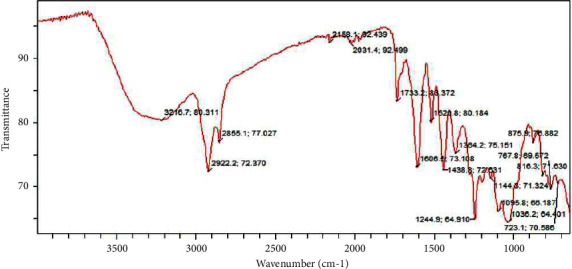
FTIR spectra of ethyl acetate fraction of ethanolic extract of *Ficus platyphylla* stem bark.

The numerous peaks shown in Figure 6 reveal that the ethyl acetate fraction contained a complex molecule. The peak contains a single bond area (2500–4000 cm^−1^) with a wider absorption band at 3216 cm^−1^. This indicates the presence of a hydrogen bond in the molecule. There is a sharp band at around 2922 and 2855 cm^−1^ revealing the presence of an aliphatic compound. Two peaks were observed at triple bond region (2000–2500 cm^−1^), at frequency range of 2158 cm^−1^ and 2031 cm^−1^ implying the presence of C=C bond in the form of nitrogen double bond and cumulated double bond compound and aromatic ring molecule, respectively. Regarding the double bond region (1500–2000 cm^−1^), narrow peak at about 1733 cm^−1^ attests the presence of a carbonyl compound, which could be aldehyde. A sharp bend was observed at 1606 cm^−1^ revealing an aromatic ring stretch and aromatic nitro compounds present, respectively. Same observation was noticed at 1520 cm^−1^. In the fingerprint region (400–1500 cm^−1^), an aromatic compound was present at strong visible bands of 767 cm^−1^ for ortho and 816 cm^−1^ for para position on aromatic ring structure. A strong visible band was observed at 1144, 1095, and 1036 cm^−1^ revealing the presence of secondary amine, cyclic ethers, and ether-oxy compounds, respectively. Peaks at 1438 cm^−1^ 1364 cm^−1^ 1244 cm^−1^ reveal saturated aliphatic (methyl), aliphatic nitro, and aromatic ether compounds, respectively. The bands at 875 cm^−1^ and 723 cm^−1^ reveal presence of peroxides and phenyl compounds as shown on the spectra.

### 5.6. FTIR Analysis of Methanol Fraction of Ethanolic Extract of *Ficus platyphylla* Stem Bark

**Figure 7:**
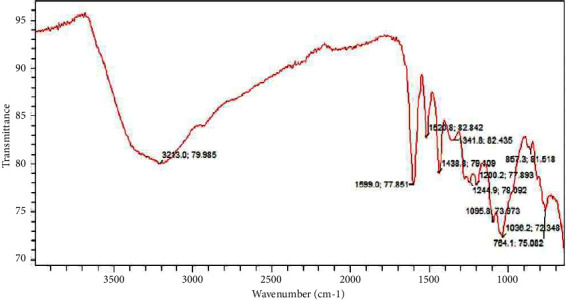
FTIR spectra of methanol fraction of ethanolic extract of *Ficus platyphylla* stem bark.

The numerous peaks shown in Figure 7 reveal that the methanol fraction contains a complex molecule. The peak contains a single bond area (2500–4000 cm^−1^), and at 3213 cm^−1^, it reveals a wider absorption band revealing a hydrogen bond in the molecule. No triple bond region (2000–2500 cm^−1^) was detected, implying the absence of C≡C bond in the molecule. Regarding the double bond region (1500–2000 cm^−1^), a sharp bend was observed at 1606 cm^−1^ revealing an alcohol-hydroxy compound and aromatic nitro compound present, respectively. Same observation was made at 1520 cm^−1^. In the fingerprint region (400–1500 cm^−1^), an aromatic compound was present at strong visible bands of 767 cm^−1^ for phenyl and 857 cm^−1^ for para position on aromatic ring structure. A strong visible band was observed at 1438, 1341, and 1244 cm^−1^ 1200 cm^−1^ 1095 cm^−1^ 1036 cm^−1^. This revealed saturated aliphatic group (methyl and methyne), aromatic ethers, aromatic ring, cyclic ether, and cyclo-hexane ring structures, respectively.

### 5.7. FTIR Analysis of Petroleum Ether Fraction of Ethanolic Extract of *Ficus platyphylla* Stem Bark

**Figure 8:**
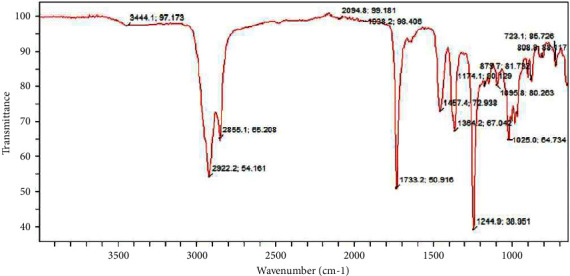
FTIR spectra of petroleum ether fraction of ethanolic extract of *Ficus platyphylla* stem bark.

The numerous peaks shown in Figure 8 reveal that the petroleum ether fraction contains a complex molecule. The peak contains a single bond area (2500–4000 cm^−1^) and reveals the presence of an ether and oxy compound in the molecule. There is a sharp band at around 2922 and 2855 cm^−1^ revealing the presence of an aliphatic compound (methyl and methylene). No triple bond region (2000–2500 cm^−1^) was detected, implying the absence of C≡C bond in the molecule, although transition metal carbonyl was observed at band of 2094 cm^−1^. Regarding the double bond region (1500–2000 cm^−1^), a narrow peak at about 1733 cm^−1^, reveals a carbonyl compound, which could be an aldehyde and at 1938 cm^−1^, informs presence of aromatic ring. In the fingerprint region (400–1500 cm^−1^), bands of 1454 cm^−^1, 1364 cm^−1^, and 1174 cm^−1^ reveal saturated aliphatic (methylene), aliphatic nitro compounds, and aromatic rings. 1244 cm^−1^peak, 1095 cm^−1^peak, and 1025 cm^−1^peak reveal an aromatic ethers, cyclic ethers, and ether-oxy compounds while 879 cm^−1^peak, 808 cm^−1^peak, and 723 cm^−1^peak reveals a peroxides, aromatic ring, and phenyl compounds, respectively.

### 5.8. FTIR Analysis of Chloroform Fraction of Ethanolic Extract of *Ficus platyphylla* Stem Bark

**Figure 9:**
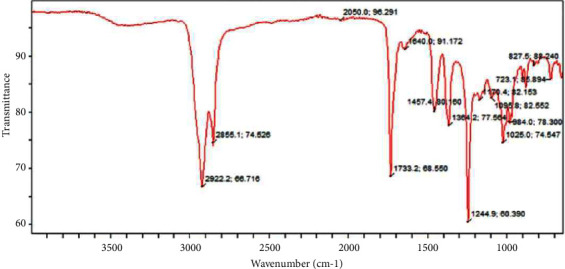
FTIR spectra of chloroform fraction of ethanolic extract of *Ficus platyphylla* stem bark.

The chloroform fraction contains complex molecule as shown by the numerous peaks. The peaks shown in Figure 9 contain a single bond area (2500–4000 cm^−1^) and reveal the presence of aliphatic chains (methyl and methylene) at 2922 cm^−1^ and 2855 cm^−1^, respectively. No triple bond region (2000–2500 cm^−1^) was detected, implying the absence of C≡C bond in the molecule, although transition metal carbonyl was observed at band of 2050 cm^−1^. Regarding the double bond region (1500–2000 cm^−1^), aldehyde and ether-oxy compound were present at peaks of 1733 cm^−1^ and 1640 cm^−1^, respectively. In the fingerprint region (400–1500 cm^−1^), carbonate ion was present at strong visible band of 1457 cm^−1^. At bands of 1364 cm^−1^, 1244 cm^−^1, and 1170 cm^−1^, aliphatic nitro compounds, aromatic ethers, and alcohol-hydroxy compound were present. A strong visible band was observed at 1095 cm^−1^, 1025 cm^−1^, 984 cm^−1^, 827 cm^−1^, and 723 cm^−1^ revealing cyclic ether, ether-oxy compounds, phosphate ion, aromatic ring, and phenyl component in the molecule.

## 6. Discussion

Plants are made up of various chemical constituents which are reported to be biologically active and are responsible for exhibiting ranges of pharmacological activities. Much of these secondary metabolites present in plant are sources of natural antioxidants with reported safety level over synthetic ones [23]. The radical scavenging activity of each fraction against DPPH and NO radicals was statistically significant at *P* < 0.05, with methanol fraction showing a strong antioxidant capacity compared to ethyl acetate, petroleum ether, and chloroform in respect to their IC_50_ values. It was observed that the antioxidant capacity of each fraction occurs in a dose-dependent manner in proportion to increasing concentration. The reducing power assay shows the activity of methanol fraction to reduce Fe^3+^ to Fe^2+^ as shown by increase in absorbance of the reaction mixture in proportion to increase in concentration. The high total flavonoid and total phenolic content of methanol fraction is evident that the fraction contained hydroxyl groups that confers sufficient antioxidant activity against DPPH and NO radicals which is in accordance to the report made by Hassan et al. [24].

The study utilized the previous analytical techniques to identify different compounds present in the respective fractions on the basis of the nature of solvent as well as nature of target compound. The LC-MS investigation of methanol fraction reveals the presence of thirteen compounds and three compounds in ethyl acetate fraction, respectively, as shown in Tables 5 and 6. However, a flavonoid identified as astilbin was present in the methanol fraction and has been established to possess certain biological functions ranging from antioxidant, antifungal, anti-carcinogen, and anticonvulsant properties [25].

The petroleum ether fraction analyzed via GC-MS identified thirty-four compounds as listed in Table 7 with trans-13-octadecenoic acid being the main compound 38.07% and cis-vaccenic acid being the least compound 0.10%. One of the identified phytochemicals, *n*-hexadecenoic acid has been reported in a previous study to possess an antioxidant, antibacterial, and antifungal property [26, 27]. 9, 12-Octadecadienoic acid was reported to possess anti-inflammatory and antibacterial properties and also used in beauty and skin care products [28]. Squalene has antioxidant, chemo-preventive activity against colon cancer, and anti-inflammatory properties [26, 27]. To elaborate further, hexadecenoic acid is found mostly in plants, animals, or micro-organism as a form of saturated fatty acid. It is used as release agents, soap production, and cosmetics. Methyl esters are found in pheromones and essential oils and are also used as fragrance [29]. Oleic acid and tripalmitin have anticholesterolemic, anti-inflammatory, antifungal, antioxidative, and antibacterial properties [30]. Another compound of importance identified is Cis-vaccenic acid. This is an omega 7 fatty acid reported to decrease LDL-cholesterol and improve insulin sensitivity [31]. In addition, 9,12-octadecadienoic acid (Z,Z) methyl ester is a potent antioxidant that helps in prevention of prostate cancer disease, Alzheimer disease, and cardiovascular diseases [31].

The chloroform fraction revealed eighteen compounds, as shown in Table 8 with 12-Oleanen-3-yl acetate (3.alpha.) being the main compound 49.25% and beta-sitosterol being the least compound 0.07%. Beta-sitosterol acts in declining the passage of cholesterol content in the blood vessels through the inhibition of cholesterol absorption at the digestive track. It is also very essential in other body processes due to its anti-inflammatory properties and improves kidney functions. Lanosterol is utilized to alleviate lens opacity in age-related cortical cataract [32]. The compounds identified in Table 7 have common biological activity which include anti-inflammatory, anti-bacterial, anti-fungal, anti-oxidant, anti-coronary, anti-acne, and anti-eczemic properties [33].

The FTIR analysis of ethyl acetate fraction contains compound with a hydroxyl group, an aromatic ring, a long saturated aliphatic chain, nitro compounds, a double bond, absence of triple bond, an aldehyde, ethers, and peroxide components. The methanol fraction contained a hydroxyl group, an aromatic ring, a long saturated aliphatic chain, absence of triple bond and ethers, and related components. The petroleum ether fraction contained ether, aromatic ring, a long aliphatic chain (methyl and methylene), absence of triple bond, a phenyl, carbonyl, nitro, and peroxide components. The chloroform fraction contained a long aliphatic chain (methyl and methylene), a carbonyl, phenyl, ether-oxy and an aromatic ring in the molecule. The previous correlations were made in correspondence to the frequency range and functional group assignment reported by Nandiyanto et al. [34].

## 7. Conclusion

The search for lead compounds from natural sources in the management of several pathological conditions is endless. The outcome of this study reveals the antioxidant capacity of the respective fractions analyzed, as well as possible compounds present, whose bioactivity has been elucidated and reported and some to be scientifically exploited in the future. It is established in this study that the methanol fraction contained poly-hydroxyl compounds compared to the other fractions analyzed, which must have contributed to its high antioxidant activity, thus making it a phytopharmacological agent to study other biological conditions such as aging, neurodegenerative disorders, cancer, and diabetes.

## Figures and Tables

**Table 1:** Phytochemical compounds of the aqueous ethanolic extract of FPSB.

Constituent	Qualitative analysis
Saponins	+
Tannins	+
Alkaloids	+
Flavonoids	+
Cardiac glycosides	+
Phenols	+
Steroids	+
Anthraquinones	−
Terpenoids	−

Presence (+), Absence (−).

**Table 2:** Percentage yield (%w/w), total phenolic, and flavonoid content of the different fractions obtained from ethanolic extract of *Ficus platyphylla* stem bark.

Plant extract	Initial weight of dried *Ficus platyphylla* stem bark = 1000 g			
	Weight of recovered extract (g)	Percentage yield (% w/w)	Total flavonoid content (mg of QE/g dry weight of FPSB fraction)	Total phenol content (mg of GAE/g dry weight of FPSB fraction)
FPSB crude extract	116.38	11.63	—	—
Petroleum ether	3.43	2.94	126.49 ± 0.004	7.02 ± 0.0145
Chloroform	2.68	2,30	274.89 ± 0.0213	59.31 ± 0.0004
Ethyl acetate	4.59	3.94	512.74 ± 0.0120	86.63 ± 0.0001
Methanol	81.04	69.63	527.38 ± 0.0040	92.46 ± 0.0003

Values are expressed as mean ± SD of triplicate determination. Values are not statistically significant at *P* *>* 0.05. GAE- gallic acid equivalent, QE- quercetin equivalent, FPSB- *Ficus platyphylla* stem bark.

**Table 3:** The percentage nitric oxide radical scavenging activity obtained from different fractions of ethanolic extract of *Ficus platyphylla* stem bark.

Concentration of fractions of *Ficus platyphylla* stem bark (*μ*g/mL)	% NO inhibition activity of different fractions of *Ficus platyphylla* stem bark				
	Ethyl acetate	Methanol	Petroleum ether	Chloroform	Ascorbic acid
20	80.14 ± 0.04^a^	92.42 ± 0.08^a^	81.64 ± 0.02^a^	77.60 ± 0.04^a^	94.32 ± 0.03^a^
40	79.46 ± 0.08^b^	87.24 ± 0.07^b^	80.06 ± 0.07^a^	75.63 ± 0.04^a^	90.22 ± 0.04^a^
60	72.84 ± 0.07^a^	84.63 ± 0.05^ab^	75.95 ± 0.05^a^	71.02 ± 0.04^a^	84.39 ± 0.07^c^
80	71.228 ± 0.0^c^	81.26 ± 0.09^c^	71.46 ± 0.02^a^	70.26 ± 0.06^b^	82.84 ± 0.10^ab^
100	68.64 ± 0.05^bc^	77.88 ± 0.03^c^	68.52 ± 0.03^a^	69.62 ± 0.06^ab^	80.32 ± 0.04^b^
200	63.99 ± 0.04^c^	73.01 ± 0.09^bc^	61.38 ± 0.07^a^	67.24 ± 0.09^c^	78.46 ± 0.07^c^
400	61.34 ± 0.03^d^	68.46 ± 0.04^bcd^	57.90 ± 0.03^b^	61.92 ± 0.04^c^	72.78 ± 0.05^d^

Values are expressed as mean ± SD of triplicate determination. Values bearing different superscript are statistically significant at *P* < 0.05 (Tukey's multiple comparison test).

**Table 4:** The reducing power activity obtained from different fractions of ethanolic extract of *Ficus platyphylla* stem bark.

Concentration of fractions of *Ficus platyphylla* stem bark (*μ*g/mL)	Reducing power (RP) activity of different fractions of *Ficus platyphylla* stem bark compared with ascorbic acid standard.				
	Ethyl acetate	Methanol	Petroleum ether	Chloroform	Ascorbic acid
20	0.32 ± 0.03	0.33 ± 0.01	0.28 ± 0.02	0.21 ± 0.01	0.34 ± 0.02
40	0.36 ± 0.02	0.38 ± 0.02	0.31 ± 0.01	0.23 ± 0.01	0.39 ± 0.03
60	0.44 ± 0.04	0.42 ± 0.04	0.33 ± 0.04	0.27 ± 0.01	0.45 ± 0.01
80	0.49 ± 0.01	0.46 ± 0.01	0.38 ± 0.02	0.28 ± 0.01	0.46 ± 0.03
100	0.50 ± 0.02	0.54 ± 0.02	0.42 ± 0.01	0.31 ± 0.01	0.72 ± 0.02
200	0.72 ± 0.03	0.99 ± 0.1	0.72 ± 0.01	0.58 ± 0.01	0.84 ± 0.01
400	0.90 ± 0.06	1.33 ± 0.03	0.88 ± 0.03	0.77 ± 0.02	1.24 ± 0.04

Values are expressed as mean ± SD of triplicate determination.

**Table 5:** LC-MS profile of compounds identified from methanol fraction obtained from ethanolic extract of *Ficus platyphylla* stem bark.

Retention time (min)	Max. intensity (intensity)	% area	Base peak (m/*z*)	Chemical composition	Compound
1.750	1835.64	0.35	196.05	C_11_H_17_NO_2_	2,4-Dimethoxyamphetamine
1.932	2647.32	0.22	214.47	C_14_H_15_NO	4-Phenoxyphenethylamine
2.622	1328.79	0.20	219.46	C_15_H_22_O	2-Octylphenylketone
4.986	2032.79	0.71	274.51	C_16_H_35_NO_2_	Ethanol, 2,2-(dodecylamino) bis-
5.752	1407126.77	1.80	289.12	C_20_H_16_O_2_	Triphenylacetic acid
9.571	7224.28	0.65	367.22	C_21_H_31_FO_4_	Methylmalonic acid, 2-fluorophenyloctylester
9.831	158583.73	0.65	390.38	C_19_H_23_F_4_NO_3_	Isonipecotic acid, N-(4-fluoro-2-trifluoromethylbenzoyl)-, pentyl ester
11.329	214827.62	94.78	418.39	C_24_H_39_NO_3_	Sarcosine, N-(4-ethylbenzoyl)-dodecylester
12.755	3232.84	0.65	451.00	C_21_H_22_O_11_	Astilbin
14.948	3361.58	0.47	461.54	C_29_H_48_O_4_	Succinic acid, 3-ethylphenyl heptadecyl ester
15.045	5352.51	0.08	500.01	C_36_H_66_	Triacontylbenzene
15.926	3892.86	1.96	599.79	C_28_H_62_O_9_Si_2_	Octaethylene glycol, 2TBDMS derivative
17.364	6738.94	0.64	601.34	C_30_H_24_N_4_O_10_	Nicofuranose

NIST standard reference database number 69.

**Table 6:** LC-MS profile of compounds identified from ethyl acetate fraction obtained from ethanolic extract of *Ficus platyphylla* stem bark NIST standard reference database number 69.

Retention time (min)	Max. intensity (intensity)	% area	Base peak (*m*/*z*)	Chemical composition	Compound
1.640	1465.86	0.23	199.51	C_10_H_11_ClO_2_	Butanoic acid, 4-chlorophenyl ester
1.824	2937.48	0.34	475.60	C_30_H_50_O_4_	Succinic acid, 3-ethylphenyl octadecyl ester
2.426	1316.62	0.42	599.09	C_36_H_78_O_2_Si_2_	1,2-Triacontanediol, di-TMS

**Table 7:** GC-MS profile of petroleum ether fraction of *Ficus platyphylla* stem bark ethanolic extract.

Peak number	Compound	Molecular formula	Retention time	Peak area (%)	Quality
1	*n*-Decanoic acid	C_10_H_20_O_2_	6.897	0.36	96
2	*n*-Hexadecanoic acid	C_16_H_32_O_2_	8.803	0.98	97
3	1,2-Benzenedicarboxylic acid, bis(2-methylpropyl) ester	C_16_H_22_O	9.247	0.32	80
4	Benzenesulfonothioic acid, S-phenyl ester	C_12_H_10_O_2_S_2_	9.500	0.81	95
5	Hexadecanoic acid, methyl ester	C_17_H_34_O_2_	11.359	0.37	94
6	Dibutyl phthalate	C_16_H_22_O_4_	11.546	1.18	97
7	*n*-Hexadecanoic acid	C_16_H_22_O_2_	12.368	14.29	99
8	9,12-Octadecadienoic acid (Z,Z)-methyl ester	C_18_H_32_O_2_	12.893	0.72	97
9	Trans-13-Octadecenoic acid, - methyl ester	C_19_H_36_O_2_	13.162	0.74	99
10	*n*-Hexadecanoic acid	C_16_H_22_O_2_	14.589	3.87	99
11	9,17-Octadecadienal, (Z)		14.787	0.49	95
12	trans-13-Octadecenoic acid, -methyl ester	C_19_H_36_O_2_	14.975	0.93	96
13	Trans-13-octadecenoic acid	C_18_H_34_O_2_	15.973	38.07	99
14	Octadecanoic acid	C_18_H_36_O_2_	16.384	4.81	99
15	Oleic acid	C_18_H_34_O_2_	17.110	2.16	98
16	1,4-benzenedicarboxylic acid, mono(1-methylethyl) ester	C_11_H_12_O_4_	22.959	0.33	30
17	cis-Vaccenic acid	C_18_H_34_O_2_	23.845	0.10	83
18	9-Octadecenal, (Z)-	C_18_H_34_O	24.056	0.17	55
19	9-Octadecenal, (Z)-	C_18_H_34_O	24.287	0.13	64
20	Cis,cis-7,10-Hexadecadienal	C_16_H_28_O	25.152	1.53	59
21	9-Octadecenoic acid (Z)-, 2-hydroxy-1-(hydroxymethyl)ethyl ester	C_21_H_40_O_4_	25.443	0.50	95
22	Octadec-9-enoic acid	C_18_H_34_O_2_	25.751	0.96	51
23	6-Octadecenoic acid, (Z)-	C_18_H_34_O_2_	26.309	1.99	66
24	9-Octadecenoic acid, (E)-	C_20_H_38_O_2_	27.117	5.05	58
25	Oleic acid	C_18_H_34_O_2_	27.230	1.04	53
26	Squalene	C_30_H_50_	27.347	1.29	64
27	9-Octadecenoic acid (Z)-, 2-hydroxy-1-(hydroxymethyl)ethyl ester	C_21_H_38_O_4_	27.955	5.20	92
28	9-Octadecenoic acid (Z)-2,3-dihydroxypropyl ester	C_21_H_40_O_4_	28.529	3.01	93
29	9-Octadecenoic acid (Z)-, 2,3-dihydroxypropyl ester	C_21_H_40_O_4_	28.730	1.33	91
30	9-Octadecenoic acid (Z)-, 2-hydroxy-1-(hydroxymethyl)ethyl ester	C_21_H_38_O_4_	29.339	2.99	94
31	Octadecanoic acid, 3-[(1-oxohexadecyl)oxy]-2-[(1-oxotetradecyl)oxy]propyl ester	C_51_H_98_O_6_	29.647	1.72	16
32	Tripalmitin	C_51_H_98_O_6_	30.132	0.70	14
33	*n*-Hexadecanoic acid, methyl(tetramethylene)silyl ester	C_21_H_42_0_2_Si	30.524	0.55	10
34	9-Octadecenoic acid (Z)-, 2-hydroxy-1-(hydroxymethyl)ethyl ester	C_21_H_40_O_4_	35.207	1.33	90

**Table 8:** GC-MS profile of chloroform fraction of *Ficus platyphylla* stem bark ethanolic extract.

Peak number	Compound	Molecular formula	Retention time	Peak area (%)	Quality
1	Beta-sitosterol	C_29_H_50_O	21.127	1.57	95
2	Gamma-sitosterol	C_29_H_50_O	21.235	1.28	99
3	Oleic acid	C_18_H_34_O_2_	22.955	0.07	56
4	Z,Z-4,16-Octadecadien-1-ol acetate	C_20_H_36_O_2_	23.581	0.81	43
5	9,19-Cyclolanost-24-en-3-ol (3.beta.)-	C_32_H_52_O_2_	25.169	2.26	56
6	9-Octadecenoic acid (Z)-2-hydroxy-1-(hydroxymethyl)ethyl ester	C_21_H_40_O_4_	25.730	0.14	86
7	9-Octadecenoic acid (Z)-2,3-dihydroxypropyl ester	C_21_H_40_O_4_	27.242	2.18	93
8	3,4-Octadiene-2,2,7,7-tetramethyl	C_12_H_22_	27.585	1.98	53
9	Olean-12-en-3-ol, acetate (3.beta.)-	C_32_H_52_O_2_	28.165	11.34	99
10	Lanosterol, TMS derivative	C_33_H_58_OSi	28.342	3.60	52
11	12-Oleanen-3-yl acetate, (3.alpha.)-	C_32_H_52_O_2_	30.216	49.25	93
12	Lup-20(29)-en-3-ol, acetate (3.beta.)-	C_30_H_50_O	30.333	21.65	99
13	Cholesta-8,24-dien-3-ol, (3.beta., 5.alpha.)-, TMS derivative	C_29_H_48_O	30.724	0.57	90
14	Octadecanoic acid, 2-[(1-oxohexadecyl)oxy]-1-[[(1-oxohexadecyl)oxy]methyl]ethyl ester	C_53_H_102_O_6_	32.601	0.38	22
15	n-propyl-11-octadecenoate	C_21_H_40_O_2_	33.058	0.45	46
16	9-Octadecenoic acid, 2-(octadecyloxy)ethyl ester	C_38_H_74_O_3_	33.440	0.77	10
17	11,13-Dimethyl-12-tetradecen-1-ol acetate	C_18_H_34_O_2_	33.861	0.95	60
18	9-Octadecene-1-[3-(octadecyloxy)propoxy]-, (Z)-	C39H_78_O_2_	34.285	0.74	81

## Data Availability

All data used are available in the manuscript.
